# An RNAi Therapy That Attenuates Multi-Organ Viremia and Improves Animal Survival in a Lethal EMCV Challenge Model

**DOI:** 10.3390/v17091240

**Published:** 2025-09-14

**Authors:** Yaxin Zhang, Jiayu Yue, Bei Wu, Jingying Xie, Jiying Xu, Wenqing Gao, Ruofei Feng, Adi Idris

**Affiliations:** 1 Engineering Research Center of Key Technology and Industrialization of Cell-Based Vaccine, Ministry of Education, Biomedical Research Center, Northwest Minzu University, Lanzhou 730030, China; 2 Gansu Tech Innovation Center of Animal Cell, Biomedical Research Center, Northwest Minzu University, Lanzhou 730030, China; 3 Key Laboratory of Biotechnology and Bioengineering of State Ethnic Affairs Commission, Biomedical Research Center, Northwest Minzu University, Lanzhou 730030, China; 4 School of Life Sciences and Engineering, Northwest Minzu University, Lanzhou 730030, China; 5 Centre for Immunology and Infection Control, School of Biomedical Sciences, Queensland University of Technology, Kelvin Grove, QLD 4702, Australia

**Keywords:** EMCV, siRNA, RNAi, nanoparticles

## Abstract

Encephalomyocarditis virus (EMCV) is an important zoonotic pathogen that infects many animals with mild symptoms. However, swine is the most receptive host and causes acute and lethal myocarditis and/or encephalitis, and induces sudden death in piglets. There are currently no approved antivirals against EMCV. In recent years, antiviral therapies based on small interfering RNA (siRNA) have been rapidly developed as effective alternative therapies. In this study, we designed siRNAs targeting highly conserved regions in the EMCV genome coinciding with VP2 and 3C genes. We show that these siRNAs were non-immunostimulatory and significantly inhibited EMCV replication in vitro. The siRNAs were then complexed in liposomes before testing in a lethal EMCV mouse model in vivo. Both prophylactic and therapeutic intravenous delivery of siRNAs ameliorated viral infection in multiple organs and improved animal survival. This is the first demonstration of the use of a liposomal delivery platform to deliver highly conserved anti-EMCV siRNAs for EMCV antiviral therapy in vivo.

## 1. Introduction

Encephalomyocarditis virus (EMCV) is an important veterinary pathogen with a wide host range. EMCV is a single-stranded positive-sense RNA picornavirus, and its genome encodes a large polyprotein, which is cleaved to produce thirteen structural and non-structural proteins. Notably, EMCV infection causes severe myocarditis and/or encephalitis in pigs with a mortality rate close to 100% in pre-weaning piglets [1]. EMCV has the capacity to spread endemically in pig farms, contributing to a significant burden on the global swine industry. Importantly, EMCV carries a strong zoonotic potential to transmit to humans via a fecal–oral transmission route [2]. Since its discovery in the late, we have yet to see an approved direct-acting antiviral therapy for EMCV infections in veterinary practice.

RNA interference (RNAi) technology utilizing small interfering RNAs (siRNAs) is an emerging new class of direct-acting antivirals. siRNAs are 21–25-base-pair, double-stranded RNA molecules that trigger RNA interference (RNAi), a highly conserved, sequence-specific post-transcriptional gene-silencing pathway in host cells. Unlike traditional approaches that often disrupt host–virus interactions, RNAi leverages the cell’s endogenous machinery to degrade specific viral RNA sequences in a virally infected cell, minimizing off-target effects. This mechanism has demonstrated success in combating RNA viruses, including human immunodeficiency virus (HIV), hepatitis C, SARS-CoV-2, and influenza, where siRNA-based therapies have shown the ability to suppress viral replication by silencing conserved genomic regions [3]. RNAi presents unique opportunities to target essential viral components from replication enzymes to structural proteins, halting infection at multiple stages of its life cycle.

Multiple EMCV targets that are amenable to RNAi silencing have been tested prophylactically in vivo but utilize either viral or plasmid DNA (pDNA)-mediated RNAi delivery systems [4,5,6]. This prophylaxis approach may be an ideal approach to protect animals from mortality due to viral encephalitis. Although viral vectors are highly efficient delivery vectors, they carry immunogenicity risks and could result in chromosomal integration events in the host [7]. Despite promising in vivo prophylaxis using viral or plasmid DNA (pDNA)-mediated RNAi delivery systems, safety concerns persist regarding immunogenicity and insertional mutagenesis risks associated with viral vectors. Non-viral delivery systems, such as lipid nanoparticles (LNPs), are being explored to mitigate these issues, though their efficacy for EMCV has not yet been developed. Additionally, combinatorial approaches integrating RNAi with interferon-based therapies show potential.

Here, we want to fine-tune anti-EMCV RNAi delivery by using siRNAs encapsulated in liposomal delivery systems. In our study, we demonstrate that an anti-EMCV siRNA-based therapeutic packaged in liposomes results in the attenuation of multi-organ viremia and rescues mice from a lethal EMCV infection.

## 2. Materials and Methods

### 2.1. Ethical Approval and Consent to Participate

All animal experiments were performed in compliance with relevant laws and institutional guidelines and in accordance with the ethical standards of the Declaration of Helsinki. All animal care and procedures have been performed according to protocols reviewed and approved by Northwest Minzu University animal ethics committee (Animal Ethics Approval # xbmu-sm-202471/202485).

### 2.2. Cells, Viruses, and Animals

HEK-293 cells and BHK-21 cells were cultured in DMEM supplemented with 10% new bovine serum (NBS) in 5% CO_2_ environment at 37 °C. The EMCV GS01 strain was used in this study. Female BALB/c mice (6–8 weeks old, ~20 g) (Lanzhou Veterinary Research Institute, Lanzhou, China) were used to assess in vivo antiviral siRNA efficacy, with each experimental group consisting of *n* = 6–8 mice.

### 2.3. siRNAs and Nucleic Acids

SiRNAs (Table 1) were designed against conserved regions of the EMCV genome, and all siRNAs are synthesized by RIBOBIO Co. (Guangzhou, China). SiRNAs were not chemically modified. Poly I:C was obtained from Invivogen (San Diego, CA, USA).

### 2.4. Antiviral siRNA In Vitro Screening and Immunostimulation

HEK-293 cells seeded overnight were treated with siRNA complexed in Lipofectamine 2000 transfection reagent (1:1.25 ratio, Invitrogen, Carlsbad, CA, USA) in Opti-MEM media (Invitrogen) before infecting with EMCV at a dose of 100 TCID_50_ 24 h. Scrambled siRNA, si-NC, was used as a negative control. After 16 h of transfection, the transfected HEK-293 cells were infected with the EMCV GS01 strain virus with 100 TCID_50_/well for 24 h. To check the immunostimulatory activity of siRNAs, HEK293 cells were either transfected with siRNA or poly I:C (positive control) for 16 h before extracting RNA to assess inflammatory cytokine mRNA expression by qPCR (IFNγ, IL-6, ISG15, and TNFα).

### 2.5. Immunoblotting

Cells harvested after 24 h of EMCV infection were lysed in radio immunoprecipitation assay (RIPA) buffer (Solarbio, Beijing, China) and subjected to sodium dodecyl sulfate polyacrylamide gel electrophoresis (SDS-PAGE) before proteins were transferred onto a polyvinylidene Fluoride Membrane (PVDF) membrane (Millipore, Belington, MA, USA). The blots were either probed with a mouse monoclonal antibody against EMCV VP1 protein (Gene Create, Wuhan, China) or a rabbit monoclonal antibody to β-actin (Abcam, Cambridge, UK) before visualizing blots using an ECL reagent (Bio-Rad, Hercules, CA, USA).

### 2.6. Quantitative PCR (qPCR)

Cells were harvested after 24 h of EMCV infection, and total RNA was extracted using Trizol reagent. RNA samples were then converted into cDNA by reverse transcription before measuring IFN-γ, IL-6, ISG15, and TNF-α mRNA expression using a SYBR Green assay (Accurate Biology, Wuhan, China). For measuring viral genomic load, cell supernatant was subjected to three rounds of freeze–thawing before extracting viral RNA in Trizol reagent. For mouse organs, the brain, heart, spleen, and kidney of mice in each group were collected, and RNA was extracted by Trizol reagent post-homogenization before measuring EMCV viral load by qPCR using EMCV-specific primers as previously carried out.

### 2.7. 50% Tissue Culture Infective Dose (TCID_50_)

TCID_50_ assays were used to measure infectious virus titres. Infected cells were subjected to three rounds of freeze–thaw cycles, and the sample clarified by centrifugation before the supernatant was collected. Supernatants were serially diluted 10-fold before exposing a monolayer of BHK-21 cells in 96-well plates. EMCV titres were calculated by the Reed and Muench method.

### 2.8. Polyethyleneimine (PEI)-siRNA Preparation

PEI (Merck #408719, Darmstadt, Germany) powder was prepared as a 1 mg/mL solution in sterile water. A final concentration of 1 mg/kg/dose (for a ~20 g mouse) of PEI-siRNA was made for every dose (PEI solution and siRNA were mixed with sterile water) and left for 20 min before performing retro-orbital intravenous injection in mice.

### 2.9. 1-Hexadecanoyl-2-(9Z-octadecenoyl)-sn-glycero-3-phospho-(1′racglycerol) (POPG) Liposome-siRNA Preparation

1-hexadecanoyl-2-(9Z-octadecenoyl)-sn-glycero-3-phospho-(1′racglycerol) (POPG) liposomes were made with 16:0–18:1 POPG (Avanti #840457P, Alabaster, AL, USA). The POPG powder was dissolved in chloroform and gently swirled until the solution formed a thin film at the bottom of the flask. An appropriate amount of siRNA was then added before the flask was shaken in a water bath at 40 °C until the thin film at the bottom of the flask was dissolved into a milky white solution (i.e., siRNA complexed in liposomes). This solution was then aspirated and extruded using a gas-tight syringe (Avanti #610017, Alabaster, AL, USA) repeatedly (up to 40 times) before collecting and storing at 4 °C. The physical characteristics of the liposomes were verified by transmission electron microscopy (TEM).

### 2.10. Transmission Electron Microscopy (TEM) Analysis of POPG Liposomes

An amount of 20 uL of resuspended siRNA-encapsulated POPG liposome samples was added dropwise to 200-mesh grids and incubated at room temperature for 10 min. The grids were then negatively stained with 2% phosphotungstic acid for 3 min, and the remaining liquid was removed using a filter paper before observing liposomes with a JEM1400 transmission electron microscope (Biomisp, Wuhan, China).

### 2.11. In Vivo Experiments

Female BALB/c mice (~20 g, 6–8-week-old) were infected by intramuscular injection with either PBS (uninfected) or 100 TCID_50_/500 μL of EMCV. PEI or POPG liposome complexed siRNAs retro-orbitally (IV) (100 μL total volume, 1 mg/kg dose) administered while under isoflurane anesthesia. Mice were monitored daily for weighing and clinical scoring.

### 2.12. Statistical Analysis

All statistical analyses were performed on GraphPad Prism 8. The data was analyzed by either Student’s *t*-test or one-way ANOVA. For all experiments, data were representative of three independent experiments with *n* = 3 technical replicates (shown as either mean ± standard deviation (SD) or mean ± standard error of mean (SEM)).

## 3. Results

### 3.1. Screening of Anti-EMCV siRNAs Targeting Multiple EMCV Genes

**Figure 1:**
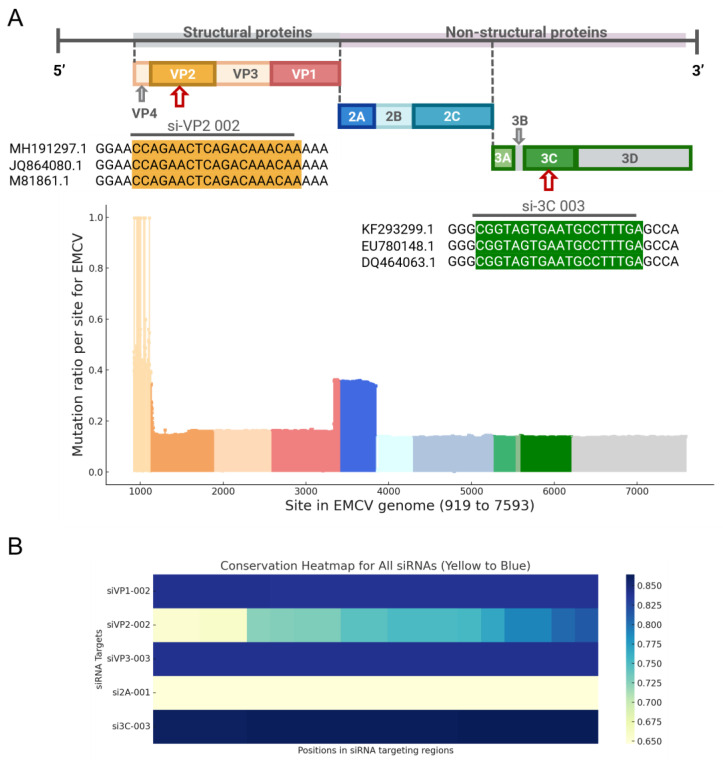
(**A**) Genome composition and mutation ratio per site for the EMCV genome, based on the analysis of 449 EMCV sequences downloaded from the NCBI Virus database. The genome structure includes both structural proteins (VP1–VP4) and non-structural proteins (2A–2C, 3A–3D). Different colors indicate variant regions across the genome, with the mutation ratio visualized for each site. Higher peaks indicate more variable regions, while lower peaks represent more conserved regions. The two best-performing siRNAs targeting VP2 (si-VP2 002, orange) and 3C (si-3C 003, green) are shown, highlighting their specific targeting regions. (**B**) Conservation heatmap for the selected siRNAs, aligned with the 449 EMCV sequences. The heatmap illustrates the conservation ratio (ranging from yellow to blue) for each siRNA across its targeting region. The siRNAs displayed are siVP1-002, siVP2-002, siVP3-003, si2A-001, and si3C-003.

**Figure 2:**
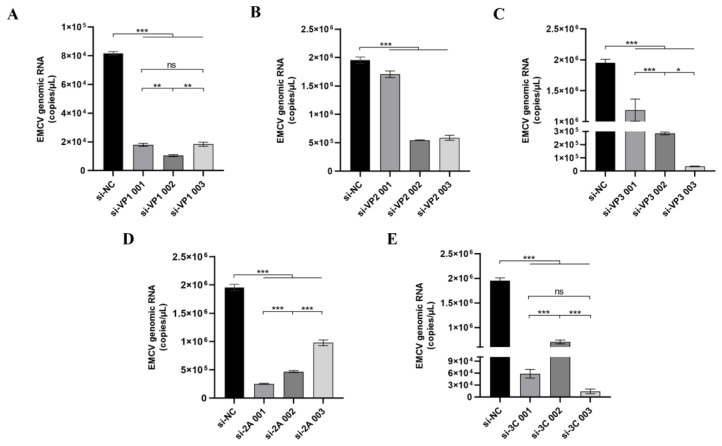
HEK293 cells were transfected with 15 nm of indicated siRNAs targeting EMCV (**A**) VP1, (**B**) VP2, (**C**) VP3, (**D**) 2A, and (**E**) 3C genes before infecting with EMCV (100 TCID_50_) 24 h later. si-NC is a non-targeting control. RNA was then extracted, and EMCV genomic RNA copy numbers determined by RT-qPCR. Data is presented as mean ± SD of three independent experiments, measured in technical duplicates. *, *p* < 0.05, **, *p* < 0.01, ***, *p* < 0.001, ns, Not Significant, Student’s *t*-test.

Analysis of 449 EMCV sequences from the NCBI Virus database revealed variability in mutation rates across the genome, with the highest mutation frequencies observed in the VP4 region (Figure 1A). Hence, we designed siRNAs against more conserved regions of the EMCV genome and tested them against a highly pathogenic EMCV lineage 1a strain, GS01. The heatmap highlights the potential efficacy of these top siRNAs, particularly si-3C, in targeting the most conserved regions across the EMCV genome (Figure 1B). We then preliminarily screened these siRNAs in vitro and selected one optimal siRNA sequence from siRNAs targeting the structural (designated as siVP1, siVP2, and siVP3) and non-structural (designated as si3C and si2A) genes. Next, the screening of the most effective siRNA was conducted through cell transfection experiments and viral copy number detection (Figure 2). For the multiple groups of siRNA designed, one optimal siRNA sequence was selected from siRNAs targeting the structural (designated as siVP1, siVP2, and siVP3) and non-structural (designated as si3C and si2A), according to the detection of viral copy number, namely siVP1-002, siVP2-002, siVP3-003, si2A-001, and si3C-003.

### 3.2. Top siRNAs Targeting Structural and Non-Structural EMCV Genes Dampen EMCV Infection In Vitro and Are Non-Immunostimulatory

**Figure 3:**
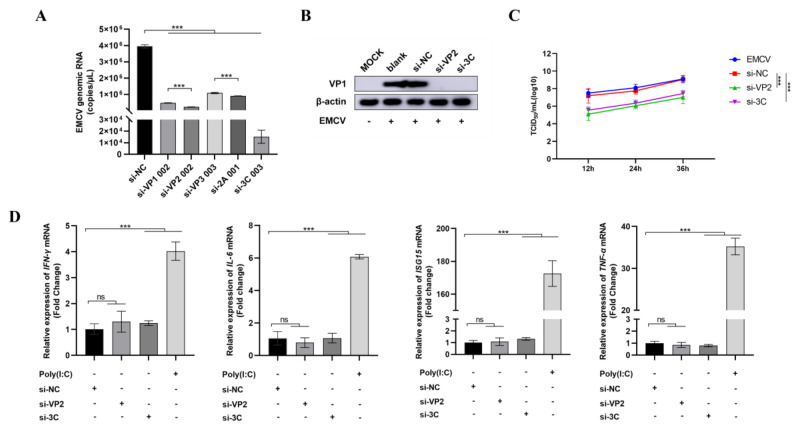
HEK293 cells were transfected with 15 nm of indicated siRNA before infecting with EMCV (100 TCID_50_) 24 h later. si-NC is a non-targeting control. (**A**) RNA was then extracted, and EMCV genomic RNA copy numbers determined by RT-qPCR. (**B**) Protein was extracted from cells, and viral knockdown was assessed by immunoblotting for EMCV VP1 protein. β-actin serves as a loading control. (**C**) After siRNA transfection, cells were infected with EMCV (100 TCID_50_) and viral titers measured at the various indicated timepoints by a TCID_50_ assay (Reed–Muench method). (**D**) HEK293 cells were either transfected with siRNA or poly I:C (positive control) for 16 h before extracting RNA to assess indicated inflammatory cytokine mRNA expression by qPCR. Data is presented as mean ± SD of three independent experiments, measured in technical duplicates. ***, *p* < 0.001, ns, Not Significant, Student’s *t*-test.

We then further screened the selected five siRNA candidates for efficient inhibition of EMCV replication in vitro and found that siRNAs targeting the structural (si-VP2) and non-structural (si-3C) genes were selected as the top anti-EMCV candidates on the basis of dampening viral genomic RNA (Figure 3A) and protein (Figure 3B) levels. Importantly, the siRNAs significantly dampened viral replication (Figure 3C), further underlining the strong antiviral activity of these siRNAs. We also confirm that these siRNAs were not immunostimulatory and hence are not responsible for the observed antiviral effect (Figure 3D).

### 3.3. Prophylactic and Therapeutic Efficacy of siRNAs Targeting EMCV VP2 and 3C in Reducing Multi-Organ Viremia and Enhancing Survival in Lethally Infected Mice

**Figure 4:**
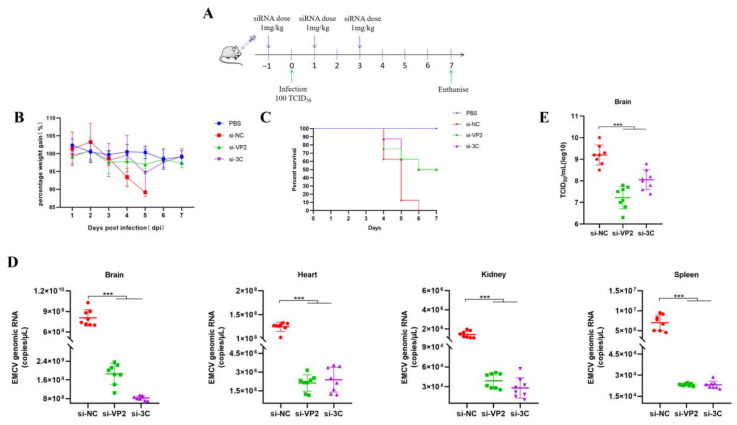
(**A**) A schematic summary of in vivo testing approach and siRNA treatment regimens. BALB/c mice were either uninfected (PBS) or infected with 100 TCID_50_ of EMCV. Mice were treated with 1 mg/kg of PEI–siRNA mixture by retro–orbital injection (IV). The control siRNA is si–NC. (**B**) Mice were weighed daily, and data points denote mean percentage weight gain. Data point is representative of the average of 8 mice ± SEM. (**C**) Probability of survival was evaluated at the indicated days post-infection (dpi). Weight loss >15% was taken as an endpoint, and mice were euthanized. Otherwise, all mice were euthanized at experimental endpoint (7 dpi). (**D**) Brain, heart, spleen, and kidney tissues were harvested to determine viral copy number by RT-qPCR. (**E**) Brain tissues were harvested to detect viral titre by TCID_50_ assay. Each data point represents one mouse. Error bars represent the SD of the data. ***, *p* < 0.001, one–way ANOVA against si–NC.

**Figure 5:**
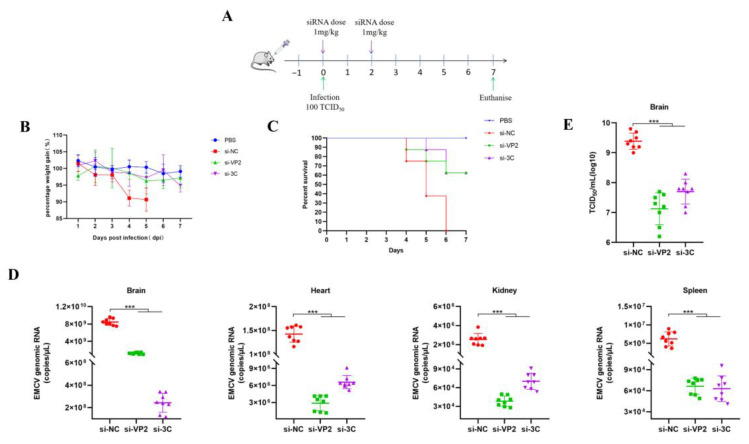
(**A**) A schematic summary of in vivo testing approach and siRNA treatment regimens. BALB/c mice were either uninfected (PBS) or infected with 100 TCID_50_ of EMCV. Mice were treated with 1 mg/kg of PEI–siRNA mixture by retro–orbital injection (IV). The control siRNA is si–NC. On day 0, siRNA was administered 2 h after EMCV infection. (**B**) Mice were weighed daily, and data points denote mean percentage weight gain. Data point is representative of the average of 8 mice ± SEM. (**C**) Probability of survival was evaluated at the indicated dpi. Weight loss > 15% was taken as an endpoint, and mice were euthanized. Otherwise, all mice were euthanized at experimental endpoint (7 dpi). (**D**) Brain, heart, spleen, and kidney tissues were harvested to determine viral copy number by RT–qPCR. (**E**) Brain tissues were harvested to detect viral titre by TCID_50_ assay. Each data point represents one mouse. Error bars represent the SD of the data. ***, *p* < 0.001, one–way ANOVA against si-NC.

SiRNAs are highly negatively charged, making it difficult for naked or unmodified siRNAs to cross the cell membrane, thus affecting cellular uptake and tissue penetration. Furthermore, siRNAs accumulating in the extracellular milieu are susceptible to degradation by nuclease and eventual clearance by the kidneys. Therefore, suitable delivery vectors are needed to efficiently transfer siRNAs into target cells. Cationic PEI is a biodegradable linear polymer that has been used for siRNA delivery for antiviral purposes [8]. To assess the antiviral activity of our in vitro validated siRNAs in vivo, PEI-complexed siRNAs targeting EMCV VP2 and 3C were administered either pre- (prophylactic) (Figure 4A) or post-exposure (Figure 5A) in a lethal EMCV infection model. We confirm the antiviral efficacy of our PEI-complexed siRNAs in vitro prior to proceeding with animal testing. EMCV can infect organs beyond the brain, including the heart, kidneys, and spleen [9], which is recapitulated in our lethal EMCV infection mouse model. Remarkably, both interventions resulted in demonstrable antiviral effects in the brain, heart, spleen, and kidneys of EMCV-infected mice (Figure 4D,E and Figure 5D,E). Moreover, irrespective of the siRNA intervention, the percentage of mouse body weight reverted almost to that of uninfected mice (Figure 4B and Figure 5B) and markedly improved clinical survival (Figure 4C and Figure 5C). Collectively, we provide evidence and proof of concept data that our anti-EMCV siRNAs can significantly reduce multi-organ viremia and rescue mice from clinical lethality.

### 3.4. Antiviral Efficacy and Survival Benefit of POPG–Based Anionic Liposome–Delivered siRNAs in EMCV–Infected Mice via a Two–Dose Regimen

**Figure 6:**
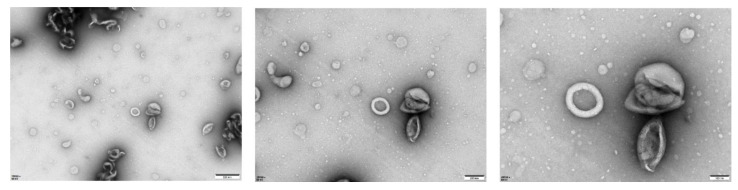
The individual siRNAs (si-3C) formulated in POPG liposomes that form large unilamellar vesicles are observed as spherical structures with a distinct electron-dense core, indicative of siRNA encapsulation. The scale bar from left to right is 500 nm, 200 nm, and 100 nm.

**Figure 7:**
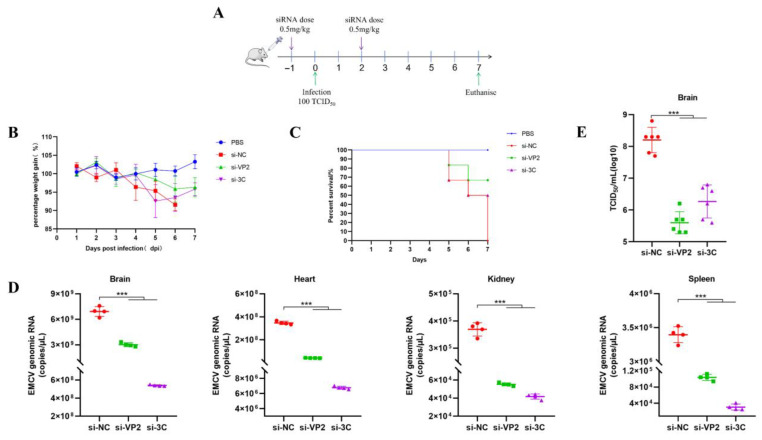
(**A**) A schematic summary of in vivo testing approach and siRNA treatment regimens. BALB/c mice were either uninfected (PBS) or infected with 100 TCID_50_ of EMCV. Mice were treated with 1 mg/kg of liposome-siRNA mixture by retro-orbital injection (IV). The control siRNA is si-NC. (**B**) Mice were weighed daily, and data points denote mean percentage weight gain. Data point is representative of the average of 8 mice ± SEM. (**C**) Probability of survival was evaluated at the indicated dpi. Weight loss >15% was taken as an endpoint, and mice were euthanized. Otherwise, all mice were euthanized at experimental endpoint (7 dpi). (**D**) Brain, heart, spleen, and kidney tissues were harvested to determine viral copy number by RT-qPCR. (**E**) Brain tissues were harvested to detect viral titre by TCID_50_ assay. Each data point represents one mouse. Error bars represent the SD of the data. ***, *p* < 0.001, one-way ANOVA against si-NC.

It is important to highlight the limitations of PEI in vivo. PEIs are not easily degraded and can contribute to organ toxicity [10]. Though several solutions have been proposed to overcome this (e.g., changing the molecular weight and structure of polymers), lipid-based formulation delivery systems are more favorable and less toxic, especially for an intravenous-based approach [11]. After decades of research, we have yet to see an approved therapeutic utilizing a PEI platform in the clinic. Considering this, we assessed another siRNA delivery approach using anionic liposomes, a safe and non-toxic platform for delivering siRNAs as opposed to cationic liposomes [12]. TEM reveals that siRNA encapsulated by POPG-based liposomes forms large unilamellar vesicles with diameters ranging between 180 and 200 nm in size (Figure 6). When mice received a prophylactic dose of liposome-siRNA before administering a final dose at two days post-EMCV infection (Figure 7A), this two-dose regimen was sufficient to dampen EMCV neuroinfection and multi-organ viremia at the experimental endpoint (Figure 7D,E). Moreover, liposome-siRNAs were sufficient to rescue mice from body weight loss and clinical lethality triggered by EMCV infection (50–60% survival for both targeting siRNAs) (Figure 7B,C), similar to that observed with PEI-siRNA (Figure 4C and Figure 5C). Overall, we show that siRNAs packaged in POPG-based liposomes could exert an appropriate antiviral effect and survival advantage in mice.

## 4. Discussion

EMCV is a zoonotic RNA virus with a broad host range spanning multiple mammalian species, including swine, mice, and potentially humans, thereby posing substantial risks to agricultural productivity and public health security [13]. Swine, as the primary natural host, are particularly vulnerable to EMCV infection, exhibiting clinical manifestations such as sudden death, severe pathological damage to vital organs, and reproductive disorders (e.g., abortion and stillbirth in sows), leading to significant economic losses in the farming industry [13]. Additionally, the detection of EMCV-specific antibodies in humans highlights its potential zoonotic transmission risk, underscoring its relevance to human biosecurity [14].

Here, we report an important finding constituting significant progress in the development of direct-acting RNA-based antivirals against an important zoonotic virus by targeting highly conserved regions in its genome. Our findings lay a good foundation for the clinical application of siRNAs as antiviral agents against veterinary viruses such as EMCV, especially in the absence of effective vaccines and antivirals. Our study presents a novel RNA interference (RNAi)-based therapeutic strategy targeting EMCV, with findings that hold important implications for advancing antiviral development. A key strength of our work lies in the rational design of siRNAs targeting highly conserved regions of the EMCV genome. Through analysis of 449 EMCV sequences from the NCBI Virus database, we identified that the VP4 region exhibits the highest mutation frequency, prompting us to focus on more conserved regions such as VP2 and 3C for siRNA design. This targeted approach addresses a critical challenge in antiviral development, which is viral genetic variability. In our study, si-VP2 and si-3C significantly reduced EMCV genomic load and viral protein expression, while also suppressing viral replication over time. Notably, these siRNAs showed no immunostimulatory activity, eliminating the possibility that their antiviral effects stem from off-target immune activation and enhancing their safety profile.

The in vivo validation of our siRNA-based strategy further reinforces its potential. Using both prophylactic and therapeutic regimens with PEI-complexed siRNAs, we observed marked reductions in multi-organ viremia (brain, heart, spleen, and kidney) in lethally infected mice. Concomitantly, treated mice showed near-restoration of body weight to uninfected levels and significantly improved survival rates. These results align with the known tropism of EMCV, which infects multiple organs beyond the brain [9], and confirm that our siRNAs effectively target viral replication across key infected tissues. However, it is essential to acknowledge the limitations of the mouse model in contextualizing clinical relevance for natural hosts like swine. A primary consideration is the differences in EMCV tropism between mice and swine. In swine, EMCV primarily causes severe myocarditis and encephalitis in pre-weaning piglets with near-100% mortality [1], whereas in our mouse model, the infection manifests as multi-organ viremia with lethality driven by systemic viral replication. Additionally, physiological differences, such as immune system maturation and organ structure, may influence the efficacy and pharmacokinetics of siRNA delivery. For example, the retro-orbital injection route used in mice may not be translatable to large-scale swine administration, and the dose (1 mg/kg) may require adjustment based on swine physiology. Future studies should therefore include swine models to validate efficacy and optimize delivery routes, ensuring that the findings can be translated to veterinary practice.

To address the safety concerns associated with PEI [8], we further evaluated an alternative delivery system using POPG liposomes. TEM analysis confirmed that siRNA-encapsulated POPG liposomes form large unilamellar vesicles with diameters of 180–200 nm, a size range conducive to cellular uptake. Our in vivo experiments using a two-dose prophylactic regimen (0.5 mg/kg per dose) demonstrated that POPG liposome-delivered siRNAs effectively reduced multi-organ viremia, rescued mice from weight loss, and achieved 50–60% survival rates. This is comparable to the efficacy of PEI-siRNA complexes. This finding is significant as anionic liposomes offer a safer alternative to cationic delivery systems, with lower toxicity and better biocompatibility [12], making them more suitable for potential clinical applications.

When placed in the context of current EMCV treatment efforts, our study fills a critical gap. Antiviral drugs targeting EMCV’s replication machinery, such as the non-nucleoside inhibitor GPC-N114, are limited by the rapid emergence of viral resistance due to mutations in the RNA-dependent RNA polymerase (RdRp) [15]. In contrast, our RNAi-based strategy targets highly conserved viral regions (VP2 and 3C), reducing the likelihood of resistance development. VP2, a structural protein, plays a key role in evading the host’s innate immune response by antagonizing the IFN-β pathway. Specifically, VP2 interacts with melanoma differentiation-associated gene 5 (MDA5), mitochondrial Antiviral Signaling Protein (MAVS), and TANK-binding kinase 1 (TBK1) through its C-terminal domain, leading to the degradation of RIG-I-like receptors (RLRs) via both proteasomal and lysosomal pathways [16]. Furthermore, VP2 inhibits signal transducer and activator of transcription 1 (STAT1) phosphorylation, a key step in the JAK-STAT signaling cascade, thereby suppressing the expression of downstream interferon-stimulated genes (ISGs), while 3C, a non-structural protease, is indispensable for viral polyprotein processing and particle assembly [1]. Its proteolytic activity, which mediates the cleavage of viral polyproteins into functional subunits, makes it an attractive target for small-molecule inhibitors. For instance, curcumol has been shown to specifically bind to the C159 active site of 3C protease, inhibiting its ability to cleave host proteins like TANK and thereby blocking viral replication [17]. By targeting these two essential proteins, our siRNAs disrupt multiple stages of the viral life cycle, enhancing their antiviral potency.

Compared to previous RNAi studies targeting EMCV, our work offers distinct advantages. Early studies used siRNAs targeting the BJC3 strain but only validated efficacy in vitro [18], while others employed viral vectors (recombinant adenoviruses) or plasmid DNA (pDNA) to deliver short hairpin RNAs (shRNAs) [4,5,6]. However, viral vectors carry risks of immunogenicity and chromosomal integration [8], and pDNA-based systems may have variable transfection efficiency. Our use of synthetic siRNAs complexed with either PEI or POPG liposomes avoids these issues, providing a safer and more scalable approach. Furthermore, the conservation of our target regions (VP2 and 3C) across EMCV isolates addresses the genetic variability observed in genes like VP1 and 2A [19,20], ensuring broader applicability of our siRNAs.

Despite these promising results, several avenues for future research remain. First, optimizing siRNA delivery systems to improve target organ specificity and stability is crucial. Lipid nanoparticles (LNPs), which have already been used in approved RNAi drugs like Patisiran [16], offer enhanced stability against nuclease degradation and targeted tissue delivery [21]. Testing LNPs for EMCV siRNA delivery could further improve efficacy and reduce off-target effects. Second, multiplexing siRNAs targeting multiple conserved viral regions could enhance antiviral potency and mitigate the risk of resistance. Finally, validating our strategy in swine models is essential to confirm its clinical relevance and guide the development of veterinary formulations.

In conclusion, our study demonstrates that liposome-delivered siRNAs targeting conserved EMCV VP2 and 3C regions effectively attenuate multi-organ viremia and improve survival in a lethal mouse model. This work provides a safe and scalable RNAi-based therapeutic approach for EMCV infection, addressing critical limitations of existing vaccination and drug strategies. While the mouse model has inherent limitations in translating to swine, our findings lay a solid foundation for further development and validation in natural hosts, with the potential to reduce the economic burden of EMCV on the global swine industry and mitigate zoonotic risks. With the advent of RNA therapeutics in the post-pandemic era, the use of RNAi technology could become the next generation of RNA medicines for veterinary practice. The use of RNAi can be applied as an emerging treatment modality against other important viruses that impact the livestock industry, including equine influenza, bovine viral diarrhea virus, and foot and mouth disease virus.

## Figures and Tables

**Table 1:** Highly conserved anti-EMCV siRNAs used in this study.

Target Gene	siRNA Name	Target Sequence
VP 1	si VP1 001	CCTTACAATTCTCCACTTT
	si VP1 002	GAAATGAGGAGACCTCAAA
	si VP1 003	GCCTGACATTAAATTCACA
VP 2	si VP2 001	GTCACAAACACCCAGTCAA
	si VP2 002	CCAGAACTCAGACAAACAA
	si VP2 003	CTGAATCTGAGAACTAACA
VP 3	si VP3 001	CAGCACAGTGCCTATTTAT
	si VP3 002	CTGGCCACCTATCAAGTGA
	si VP3 003	GCAGGCGACTTATGCGATT
2A	si 2A 001	GCGGACGTGATTCTGAGAT
	si 2A 002	CGGACCTACTGATCCATGA
	si 2A 003	GCAGAACCATGTAGAGTGA
3C	si 3C 001	CGGACATACCCATGATGTA
	si 3C 002	CGCACCCTGGCAGTAAATA
	si VP3 003	CGGTAGTGAATGCCTTTGA

## Data Availability

The original contributions presented in this study are included in the article/Supplementary Material. Further inquiries can be directed to the corresponding authors.

## Supplementary Materials

The following supporting information can be downloaded at: https://www.mdpi.com/article/10.3390/v17091240/s1, Table S1: Highly conserved anti-EMCV siRNAs used in this study.
