# Supplementary material for: An RNAi Therapy That Attenuates Multi-Organ Viremia and Improves Animal Survival in a Lethal EMCV Challenge Model
Source: Viruses. 2025 Sep 14;17(9):1240. doi: 10.3390/v17091240 (PMC12474460; doi:10.3390/v17091240)
Supplement: Supplementary file 1 [file viruses-17-01240-s001.zip › viruses-3852380-supplementary.pdf]

Table S1 Highly conserved anti-EMCV siRNAs used in this study

**Target gene**	**siRNA name**	**Target sequence**
VP1	siVP1-001	CCTTACAATTCTCCACTTT
	siVP1-002	GAAATGAGGAGACCTCAAA
	siVP1-003	GCCTGACATTAAATTCACA
VP2	siVP2-001	GTCACAAACACCCAGTCAA
	siVP2-002	CCAGAACTCAGACAAACAA
	siVP2-003	CTGAATCTGAGAACTAACA
VP3	siVP3-001	CAGCACAGTGCCTATTTAT
	siVP3-002	CTGGCCACCTATCAAGTGA
	siVP3-003	GCAGGCGACTTATGCGATT
2A	si2A-001	GCGGACGTGATTCTGAGAT
	si2A-002	CGGACCTACTGATCCATGA
	si2A-003	GCAGAACCATGTAGAGTGA
3C	si3C-001	CGGACATACCCATGATGTA
	si3C-002	CGCACCCTGGCAGTAAATA
	si3C-003	CGGTAGTGAATGCCTTTGA
